# Supplementary material for: Winners’ strategies: Comprehensive analysis and optimization of 2-point shots in 3x3 basketball using multi-criteria decision support analysis, on the example of two Olympic National Teams
Source: PLoS One. 2025 Apr 29;20(4):e0322024. doi: 10.1371/journal.pone.0322024 (PMC12040086; doi:10.1371/journal.pone.0322024)
Supplement: Attachment — Submitted filename: PONE -D-24-34221_ MA_Reviewer.docx [file pone.0322024.s001.docx]

**Overall Evaluation:**

The article presents an innovative approach to the strategic analysis of 2-point shots in 3x3 basketball, making a significant contribution to the literature in this field. The use of advanced analytical methods such as multi-criteria decision analysis (MCDA) enhances the value of the study in both academic and practical domains. When evaluated against PLOS ONE's publication criteria, the study has several strengths, including a title and abstract that reflect the content, and a methodology that is detailed and transparently presented. However, the methodological explanations need to include more information on participant selection and the ethical approval process. Additionally, the limited sample size and the lack of advanced statistical methods in data analysis introduce limitations in terms of the study's generalizability.

Presenting the results and discussion in a more concise and clearer manner would better align with PLOS ONE's policy of appealing to a broader readership. In terms of language and structure, the article generally uses scientific terminology effectively and has strong language, but the complexity of some sentences could be simplified to enhance clarity. A more comprehensive comparison with the literature in the discussion section would highlight the study’s original contribution and its alignment with another research.

Overall, the article is deemed suitable for publication in PLOS ONE, but it could be further strengthened with specific improvements. Such revisions would align with the literature emphasizing methodological clarity and transparency in data presentation within sports science, thereby enhancing the article's scientific value and the benefit it provides to the reader.

**Title & Abstract**

**Positive:** The title clearly and concisely reflects the content and focus of the study, in accordance with PLOS ONE's criteria for clarity and accessibility. It explicitly indicates that the study focuses on 2-point shot strategies in 3x3 basketball, highlighting the innovative aspect of the paper. The abstract effectively summarizes the study’s aim, methods, and key findings, presenting the main points of the research in a clear manner. This aligns with PLOS ONE’s principle of being accessible to a broad audience. Additionally, the paper addresses a topic rarely explored in the literature by focusing on the analysis and optimization of 2-point shots in 3x3 basketball, thereby providing an innovative contribution to the field of sports science. The use of methodological tools such as multi-criteria decision analysis (MCDA) broadens the scope of such studies and offers a new perspective for sports performance analysis.

**Negative:** The abstract should be shorter and more focused to meet PLOS ONE's expectations. In its current form, it contains too many technical details, which might make it challenging for readers to quickly grasp the main findings of the study. To enhance clarity, it is recommended to reduce technical details and place greater emphasis on the main findings. Furthermore, it would be beneficial to more clearly articulate how the paper differentiates itself from other studies. Specifically, better highlighting the unique dynamics of 3x3 basketball and how this study adds a new perspective to the literature would be advantageous.

**In conclusion,** the title and abstract effectively highlight the innovative aspect of the study and its contribution to sports science. However, making the abstract more concise and focused would help readers understand the main contributions more easily. Therefore, some revisions are recommended to ensure the abstract fully aligns with PLOS ONE’s criteria.

**Introduction**

**Positive:** The introduction clearly outlines the historical development of 3x3 basketball and identifies gaps in the existing literature, highlighting the study's originality and scientific contribution. The research questions are defined clearly and concisely, making it easier for the reader to understand the purpose and significance of the study.

**Negative:** Some parts of the introduction could be long and repetitive. For instance, the sentence **"The characteristics of this sport are defined by the unique dynamics and intensity of the game..."** repeats information already mentioned in the previous sentence. A more concise and focused introduction would help the reader grasp the main idea more quickly.

**Materials and Methods**

**Positive:** The methods section provides a detailed explanation of the analytical tools and procedures used. The clear description of the application of the video analysis program and MCDA enhances the study's repeatability and reliability. The level of detail in the methods section demonstrates the scientific rigor of the study.

**Negative:** More information on participant selection and the ethical approval process may be needed. **Specifically, providing further explanation regarding the validity and reliability of the video analysis would strengthen the methodology.** Additionally, it could be beneficial to include more discussion about the advantages and potential limitations of the methods used.

**Findings**

**Positive:** The results section presents the findings in a detailed and comprehensive manner. The use of tables and graphs to support the data makes it easier for the reader to understand and visualize the results. Notably, the findings highlighting the relationship between shooting strategies and game outcomes clearly demonstrate the main contribution of the study.

**Discussion**

**Positive:** The discussion section compares the findings with the existing literature, highlighting the study's contributions and originality. Statements like "This observation highlights that 2-point shooting training should be a priority for coaches and players..." clearly outline the practical applications of the research and provide recommendations for basketball coaches.

**Negative:** Some expressions in the discussion are repetitive and lengthy. For instance, sentences like "Teams with more effective players with better 2-point shooting accuracy have a greater chance of dominating the pitch" may appear to be a restatement of previous findings. Keeping the discussion more focused and concise would help convey the main conclusions more effectively.

**Conclusion**

**Positive:** The conclusion section summarizes the key findings of the research, emphasizing the strategic importance of 2-point shots in 3x3 basketball. Additionally, by offering suggestions on how these findings can be applied in practice, it demonstrates the article's contribution to the field and its potential impact. This can be valuable for coaches and sports analysts.

**Negative:** However, the conclusion could focus more on the study's limitations and the generalizability of the findings. For instance, more information could be provided on how these findings might apply to other basketball variations or different game scenarios. Additionally, suggestions on how the study could be expanded and which aspects should be explored in future research would be beneficial.

**Ethics and Data Sharing**

**Positive:** The article states that ethical standards were followed and data sharing was conducted transparently. This is crucial for the replicability of the study and for it to be re-evaluated by other researchers. Additionally, since the study did not involve human participants or animals, there are no ethical concerns in this regard.
