# Supplementary material for: Winners’ strategies: Comprehensive analysis and optimization of 2-point shots in 3x3 basketball using multi-criteria decision support analysis, on the example of two Olympic National Teams
Source: PLoS One. 2025 Apr 29;20(4):e0322024. doi: 10.1371/journal.pone.0322024 (PMC12040086; doi:10.1371/journal.pone.0322024)
Supplement: Attachment — Submitted filename: Response to Reviewers.docx [file pone.0322024.s003.docx]

**Response to Editor and Reviewers**

“PONE-D-24-34221”

“Winners' strategies: Comprehensive analysis and optimization of 2-point shots in 3x3 basketball using multi-criteria decision support analysis, on the example of two Olympic National Teams.”

We thank you for your detailed analysis and constructive comments on our article. Your comments were extremely important to us, especially since the article was rated positively. Your valuable indications allowed us to improve the manuscript and will influence our commitment to further developing our work and project basketball 3 on 3. We have looked over all suggestions and made appropriate corrections. They improved the substantive and formal aspects of the article. The changes have been marked in blue to facilitate their identification (plus the option to track changes has been enabled. We hope that the introduced corrections fully meet your expectations. Additionally, we have introduced numbering for clarity and easier finding of responses: 0 - main editor, 1 - first reviewer, 2 - second reviewer, 3 - third reviewer. Once again, thank you for your time and contribution to developing our article. I added line numbering options with the change tracking file to make it easier to find corrections.

**Overall Evaluation:**

The article presents an innovative approach to the strategic analysis of 2-point shots in 3x3 basketball, making a significant contribution to the literature in this field. The use of advanced analytical methods such as multi-criteria decision analysis (MCDA) enhances the value of the study in both academic and practical domains. When evaluated against PLOS ONE's publication criteria, the study has several strengths, including a title and abstract that reflect the content, and a methodology that is detailed and transparently presented.

(0.1) However, the methodological explanations need to include more information on participant selection and the ethical approval process. Additionally, the limited sample size and the lack of advanced statistical methods in data analysis introduce limitations in terms of the study's generalizability.

Thank you very much for your valid comments. The individual changes were introduced in the suggested subsections. (Description of the research group), (research ethics issues), (Limitations and statistical analysis).

(0.2) Presenting the results and discussion in a more concise and clearer manner would better align with PLOS ONE's policy of appealing to a broader readership.

Thank you very much for your advice. The following changes were made in the results section: unnecessary information was removed, and the entire chapter was divided into subchapters to facilitate the interpretation of results. The discussion section was also modified, considering all the reviewers' suggestions. The manuscript's language was slightly changed to be easier and more readable.

In terms of language and structure, the article generally uses scientific terminology effectively and has strong language,

(0.3) but the complexity of some sentences could be simplified to enhance clarity. A more comprehensive comparison with the literature in the discussion section would highlight the study’s original contribution and its alignment with another research.

We are very grateful for your advice. The entire work has been subjected to a renewed linguistic analysis. Sentences that were difficult to interpret have been modified, and their reception by the reader should be easier. A review of new literature on the 3 on 3 game was also made, which was included in both the introduction and discussion chapters. The uniqueness of the work was clearly emphasized.

Overall, the article is deemed suitable for publication in PLOS ONE, but it could be further strengthened with specific improvements. Such revisions would align with the literature emphasizing methodological clarity and transparency in data presentation within sports science, thereby enhancing the article's scientific value and the benefit it provides to the reader.

**Title & Abstract**

**Positive:** The title clearly and concisely reflects the content and focus of the study, in accordance with PLOS ONE's criteria for clarity and accessibility. It explicitly indicates that the study focuses on 2-point shot strategies in 3x3 basketball, highlighting the innovative aspect of the paper. The abstract effectively summarizes the study’s aim, methods, and key findings, presenting the main points of the research in a clear manner. This aligns with PLOS ONE’s principle of being accessible to a broad audience. Additionally, the paper addresses a topic rarely explored in the literature by focusing on the analysis and optimization of 2-point shots in 3x3 basketball, thereby providing an innovative contribution to the field of sports science. The use of methodological tools such as multi-criteria decision analysis (MCDA) broadens the scope of such studies and offers a new perspective for sports performance analysis.

**Negative:**

(0.4) The abstract should be shorter and more focused to meet PLOS ONE's expectations. In its current form, it contains too many technical details, which might make it challenging for readers to quickly grasp the main findings of the study. To enhance clarity, it is recommended to reduce technical details and place greater emphasis on the main findings. Furthermore, it would be beneficial to more clearly articulate how the paper differentiates itself from other studies. Specifically, better highlighting the unique dynamics of 3x3 basketball and how this study adds a new perspective to the literature would be advantageous.

We appreciate your valuable comments on the abstract. We agree that a more focused approach and reduced technical details would help readers better understand the main findings of our study. In response to your suggestions, we have shortened the abstract, reducing the number of technical details to focus on the most important conclusions of our study.

Additionally, we have clearly stated how our study differs from previous work, emphasizing the unique dynamics of 3x3 basketball and the contribution this study makes to the existing literature. We hope that these changes will improve the clarity of the abstract and meet the expectations of PLOS ONE.

**In conclusion,** the title and abstract effectively highlight the innovative aspect of the study and its contribution to sports science. However, making the abstract more concise and focused would help readers understand the main contributions more easily. Therefore, some revisions are recommended to ensure the abstract fully aligns with PLOS ONE’s criteria.

Thank you very much for your suggestion. The text of the abstract has been completely rewritten, and the new version has been placed in the manuscript.

**Introduction**

**Positive:** The introduction clearly outlines the historical development of 3x3 basketball and identifies gaps in the existing literature, highlighting the study's originality and scientific contribution. The research questions are defined clearly and concisely, making it easier for the reader to understand the purpose and significance of the study.

**Negative:**

(0.5) Some parts of the introduction could be long and repetitive. For instance, the sentence **"The characteristics of this sport are defined by the unique dynamics and intensity of the game..."** repeats information already mentioned in the previous sentence. A more concise and focused introduction would help the reader grasp the main idea more quickly.

Following the reviewer's suggestions, the introduction text was edited from the start. Repeated sentences were removed. The most important aspects of the game were included. The changes also forced an update in the bibliography. In the manuscript, the subchapter was replaced with a new version.

**Materials and Methods**

**Positive:** The methods section provides a detailed explanation of the analytical tools and procedures used. The clear description of the application of the video analysis program and MCDA enhances the study's repeatability and reliability. The level of detail in the methods section demonstrates the scientific rigor of the study.

**Negative:**

(0.6) More information on participant selection and the ethical approval process may be needed. **Specifically, providing further explanation regarding the validity and reliability of the video analysis would strengthen the methodology.** Additionally, it could be beneficial to include more discussion about the advantages and potential limitations of the methods used.

Thank you very much for pointing out the details. The corrections will positively impact the reception of the work.

1. Selection of participants and the ethical consent process: changes introduced in the text in line 264 new manuscript.

2. Reliability and credibility of the video analysis: Changes introduced from line no. 221

3. Advantages and limitations of the methods used: Additional information added from line no. 226

**Findings**

**Positive:** The results section presents the findings in a detailed and comprehensive manner. The use of tables and graphs to support the data makes it easier for the reader to understand and visualize the results. Notably, the findings highlighting the relationship between shooting strategies and game outcomes clearly demonstrate the main contribution of the study.

**Discussion**

**Positive:** The discussion section compares the findings with the existing literature, highlighting the study's contributions and originality. Statements like "This observation highlights that 2-point shooting training should be a priority for coaches and players..." clearly outline the practical applications of the research and provide recommendations for basketball coaches.

**Negative:**

(0.7) Some expressions in the discussion are repetitive and lengthy. For instance, sentences like "Teams with more effective players with better 2-point shooting accuracy have a greater chance of dominating the pitch" may appear to be a restatement of previous findings. Keeping the discussion more focused and concise would help convey the main conclusions more effectively.

Thank you for your valuable feedback on the discussion section.

In response to your suggestions, we have made the following changes:

1. We have removed repetitions and reduced the number of extended statements to clarify the main conclusions.

2. We have shortened and focused the conclusions, presenting the key points simply and concisely.

3. We have focused on the implications of the results for coaching practice and future research, avoiding unnecessary details.

We hope that the changes we have made meet your expectations and improve the readability of the paper.

**Conclusion**

**Positive:** The conclusion section summarizes the key findings of the research, emphasizing the strategic importance of 2-point shots in 3x3 basketball. Additionally, by offering suggestions on how these findings can be applied in practice, it demonstrates the article's contribution to the field and its potential impact. This can be valuable for coaches and sports analysts.

**Negative:**

(0.8) However, the conclusion could focus more on the study's limitations and the generalizability of the findings. For instance, more information could be provided on how these findings might apply to other basketball variations or different game scenarios. Additionally, suggestions on how the study could be expanded and which aspects should be explored in future research would be beneficial.

Thank you for your valuable feedback on the discussion section.

In response to your suggestions, we have made the following changes:

1. We have added a paragraph highlighting the study's limitations, such as the analysis of only two teams and the specific setting of 3x3 basketball, which may limit the generalizability of the results to other teams and basketball variants.

2. We have suggested potential directions for future research, including extending the analysis to other teams and skill levels, comparisons with 5x5 basketball, and the possibility of using advanced technologies (e.g., LPS tracking systems) to obtain additional data.

**Ethics and Data Sharing**

**Positive:** The article states that ethical standards were followed and data sharing was conducted transparently. This is crucial for the replicability of the study and for it to be re-evaluated by other researchers. Additionally, since the study did not involve human participants or animals, there are no ethical concerns in this regard.

(0.9). As we previously announced, the raw data has been placed in a public repository at DOI. 10.5281/zenodo.14021215

Reviewers' comments:

Reviewer's Responses to Questions

**Comments to the Author**

1. Is the manuscript technically sound, and do the data support the conclusions?

The manuscript must describe a technically sound piece of scientific research with data that supports the conclusions. Experiments must have been conducted rigorously, with appropriate controls, replication, and sample sizes. The conclusions must be drawn appropriately based on the data presented.

Reviewer #1: Yes

Reviewer #2: Yes

Reviewer #3: Yes

Thank you very much, all reviewers.

2. Has the statistical analysis been performed appropriately and rigorously?

Reviewer #1: Yes

Reviewer #2: I Don't Know

Reviewer #3: Yes

Thank you very much, all reviewers.

3. Have the authors made all data underlying the findings in their manuscript fully available?

The [PLOS Data policy](http://c05y1x9s.r.us-east-2.awstrack.me/L0/http:%2F%2Fwww.plosone.org%2Fstatic%2Fpolicies.action%23sharing/1/010f0191fa1b215f-d43365b5-6a3b-4aba-8376-0b36d4429c31-000000/_R7z2HH2LNtSGKMYIpiadc1vdQ4=176) requires authors to make all data underlying the findings described in their manuscript fully available without restriction, with rare exception (please refer to the Data Availability Statement in the manuscript PDF file). The data should be provided as part of the manuscript or its supporting information, or deposited to a public repository. For example, in addition to summary statistics, the data points behind means, medians and variance measures should be available. If there are restrictions on publicly sharing data—e.g. participant privacy or use of data from a third party—those must be specified.

Reviewer #1: Yes

Reviewer #2: Yes

Reviewer #3: Yes

Thank you very much, all reviewers.

4. Is the manuscript presented in an intelligible fashion and written in standard English?

PLOS ONE does not copyedit accepted manuscripts, so the language in submitted articles must be clear, correct, and unambiguous. Any typographical or grammatical errors should be corrected at revision, so please note any specific errors here.

Reviewer #1: Yes

Reviewer #2: No

Reviewer #3: Yes

Reply to reviewer #2:

After the corrections were made, the text was subjected to linguistic proofreading again; the changes are visible in the marked file.

5. Review Comments to the Author

Please use the space provided to explain your answers to the questions above. You may also include additional comments for the author, including concerns about dual publication, research ethics, or publication ethics. (Please upload your review as an attachment if it exceeds 20,000 characters)

**Reviewer #1:** The manuscript technically sound, and the data support the conclusion. Although it is well discussed, it is in much detailed detail, it could be summarized.
The authors made all data underlying the findings in their manuscript fully available.
The manuscript presented in an intelligible fashion and written in standard English.

Reply to reviewer #1:

1.1 We thank the reviewer for his positive assessment of the manuscript. We also appreciate your advice regarding the excessively detailed discussion in some parts of the article. Following your suggestion, we've revised the discussion and conclusions to make it more open and easier to digest without losing key information. After making changes to the text, we have rechecked the language. We hope that the changes are in line with what you said.

**Reviewer #2:** Introduction is in overall well-written. Maybe there is a sufficient data about physical and motor status of 3x3 players comparing to the data related to the topic.
The name of the author in the reference no. 22 is Erculj instead of Ervculj.

Reply to reviewer #2:

Thank you for the generally positive feedback. A correction in the form of a "typo" error in the name and surname of one of the authors cited in the work has been corrected. We apologize. It was by no means intentional.

**Reviewer #3:** The article presents an innovative approach to the strategic analysis of 2-point shots in 3x3 basketball, making a significant contribution to the literature in this field. The use of advanced analytical methods such as multi-criteria decision analysis (MCDA) enhances the value of the study in both academic and practical domains. When evaluated against PLOS ONE's publication criteria, the study has several strengths, including a title and abstract that reflect the content, and a methodology that is detailed and transparently presented.

(3.1) However, the methodological explanations need to include more information on participant selection and the ethical approval process.

Reply to reviewer #3.1:

We appreciate your suggestions for clarifying the selection of participants and the ethical issues related to the study. Following your suggestions, we have supplemented the methodology section with detailed information on the selection of teams (Poland and Serbia) and an explanation of the ethical aspects of the study. We have added an explanation that the analysis was based solely on publicly available video recordings, which allowed us to avoid the need for ethical approval.

We hope that the changes made fully meet your expectations.

(3.2) Additionally, the limited sample size and the lack of advanced statistical methods in data analysis introduce limitations in terms of the study's generalizability.

Reply to reviewer #3.2:

Thank you for pointing out the key limitations of our study. Following your suggestions, we have expanded the limitations section to:

1. Clearly emphasize the sample size as a limitation (analysis of only two national teams) that impacts the generalizability of the results to other teams, tournaments, and levels of competition.

2. Emphasize the need for more advanced statistical methods in future studies to obtain more precise and general conclusions.

Our study is considered a Pilot for the analysis of all teams competing in the next Olympics, both men's and women's.

(3.3) Presenting the results and discussion in a more concise and clearer manner would better align with PLOS ONE's policy of appealing to a broader readership.

Reply to reviewer #3.3:

We appreciate your valuable feedback on the conciseness and accessibility of the results and discussion sections. Following your suggestions, the relevant sections have been rewritten to make them more readable for a wider audience.

We have made the following changes:

1. We have shortened the analysis of the results, focusing on key observations and their interpretation, and replaced detailed descriptions with shorter summaries.

2. We have simplified the language, eliminating overly technical terms, to make the text more understandable for readers beyond a narrow circle of specialists.

3. We have divided the results and discussion sections into subsections, making it easier to follow the main conclusions, the significance of the results, and their practical implications.

4 We have changed the results section's presentation scheme to emphasize our study's significance further.

(3.4) In terms of language and structure, the article generally uses scientific terminology effectively and has strong language, but the complexity of some sentences could be simplified to enhance clarity.

Reply to reviewer #3.4:

We appreciate your suggestions for simplifying the language and structure of the article. In response to your comments, we have made the following changes:

1. We have simplified complex sentences by splitting long phrases into shorter and clearer formulations when possible without losing the context of the message.

2. We have also replaced specialized terms with more accessible expressions when possible while maintaining the scientific nature of the text (basketball terminology).

3. We have shortened paragraphs and divided subsections so that each part focuses on one topic, which increases the article's readability and accessibility.

(3.5) A more comprehensive comparison with the literature in the discussion section would highlight the study’s original contribution and its alignment with other research.

Reply to reviewer #3.5:

We appreciate your suggestions for expanding the discussion section.

In response to your suggestions, we have added a more detailed comparison of our study’s results to the literature, indicating both similarities and differences with previous studies. We have also highlighted unique aspects of our study to more clearly indicate its original contribution. In summary, the current paper includes 6 additional works related to the topic of 3v3 gameplay.

Additionally, we have explained the significance of the results in the context of existing research, which better places our work in the broader body of research.

We hope that the changes we have made meet your expectations.

Overall, the article is deemed suitable for publication in PLOS ONE, but it could be further strengthened with specific improvements. Such revisions would align with the literature emphasizing methodological clarity and transparency in data presentation within sports science, thereby enhancing the article's scientific value and the benefit it provides to the reader.
