# Supplementary material for: Winners’ strategies: Comprehensive analysis and optimization of 2-point shots in 3x3 basketball using multi-criteria decision support analysis, on the example of two Olympic National Teams
Source: PLoS One. 2025 Apr 29;20(4):e0322024. doi: 10.1371/journal.pone.0322024 (PMC12040086; doi:10.1371/journal.pone.0322024)
Supplement: Attachment — Submitted filename: Response to Reviewers V4.docx [file pone.0322024.s004.docx]

**Response to Editor and Reviewers**

“PONE-D-24-34221”

“Winners' strategies: Comprehensive analysis and optimization of 2-point shots in 3x3 basketball using multi-criteria decision support analysis, on the example of two Olympic National Teams.”

Thank you for your detailed analysis and constructive comments on our article. Your feedback was invaluable to us, especially since the article received a positive assessment. Your insightful suggestions allowed us to enhance the manuscript and will shape our dedication to the ongoing development of our work and the 3-on-3 basketball project. We have conducted another thorough analysis and successfully improved several sentences, enhancing both the substantive and formal aspects of the article.

The responses are highlighted in blue for easier identification (and the option to track changes is enabled in the document). We hope that the corrections made fully meet your expectations. Additionally, we have included numbering for clarity and to facilitate the location of responses: 0 - main editor.

Thank you once again for your time and contribution to the development of our article.

**Overall Evaluation:**

The article presents an innovative approach to the strategic analysis of 2-point shots in 3x3 basketball, making a significant contribution to the literature in this field. The use of advanced analytical methods such as multi-criteria decision analysis (MCDA) enhances the value of the study in both academic and practical domains. When evaluated against PLOS ONE's publication criteria, the study has several strengths, including a title and abstract that reflect the content, and a methodology that is detailed and transparently presented.

(0.1) However, the methodological explanations need to include more information on participant selection and the ethical approval process. Additionally, the limited sample size and the lack of advanced statistical methods in data analysis introduce limitations in terms of the study's generalizability.

Thank you very much for your valuable comments. The specific changes have been made in the proposed subsections: (Description of the research group), (research ethics issues), and (Limitations and statistical analysis)

(0.2) Presenting the results and discussion in a more concise and clearer manner would better align with PLOS ONE's policy of appealing to a broader readership.

Thank you very much for your advice. The following changes were made to the results section: unnecessary information was removed, and the entire chapter was divided into subsections to make the results easier to interpret. The discussion section was also modified, incorporating all the suggestions from the reviewers. Everything was included in the previous version, V3. The language of the manuscript was slightly adjusted once again to enhance clarity, accessibility, and readability. Thus, the text is now more accessible.In terms of language and structure, the article generally uses scientific terminology effectively and has strong language,

(0.3) but the complexity of some sentences could be simplified to enhance clarity. A more comprehensive comparison with the literature in the discussion section would highlight the study’s original contribution and its alignment with another research.

We sincerely appreciate your advice. The entire work has again undergone linguistic analysis V3 vs V4. Sentences that were difficult to interpret have been revised, making them easier for readers to understand. The uniqueness of the work has been clearly emphasized

Overall, the article is deemed suitable for publication in PLOS ONE, but it could be further strengthened with specific improvements. Such revisions would align with the literature emphasizing methodological clarity and transparency in data presentation within sports science, thereby enhancing the article's scientific value and the benefit it provides to the reader.

**Title & Abstract**

**Positive:** The title clearly and concisely reflects the content and focus of the study, in accordance with PLOS ONE's criteria for clarity and accessibility. It explicitly indicates that the study focuses on 2-point shot strategies in 3x3 basketball, highlighting the innovative aspect of the paper. The abstract effectively summarizes the study’s aim, methods, and key findings, presenting the main points of the research in a clear manner. This aligns with PLOS ONE’s principle of being accessible to a broad audience. Additionally, the paper addresses a topic rarely explored in the literature by focusing on the analysis and optimization of 2-point shots in 3x3 basketball, thereby providing an innovative contribution to the field of sports science. The use of methodological tools such as multi-criteria decision analysis (MCDA) broadens the scope of such studies and offers a new perspective for sports performance analysis.

**Negative:**

(0.4) The abstract should be shorter and more focused to meet PLOS ONE's expectations. In its current form, it contains too many technical details, which might make it challenging for readers to quickly grasp the main findings of the study. To enhance clarity, it is recommended to reduce technical details and place greater emphasis on the main findings. Furthermore, it would be beneficial to more clearly articulate how the paper differentiates itself from other studies. Specifically, better highlighting the unique dynamics of 3x3 basketball and how this study adds a new perspective to the literature would be advantageous.

We appreciate your valuable comments on the abstract. We agree that a more focused approach and reduced technical details would help readers better understand the main findings of our study. In response to your suggestions, we have shortened the abstract, reducing the number of technical details to focus on the most important conclusions of our study again V3 vs V4.

Additionally, we have clearly stated how our study differs from previous work, emphasizing the unique dynamics of 3x3 basketball and the contribution this study makes to the existing literature.

**In conclusion,** the title and abstract effectively highlight the innovative aspect of the study and its contribution to sports science. However, making the abstract more concise and focused would help readers understand the main contributions more easily. Therefore, some revisions are recommended to ensure the abstract fully aligns with PLOS ONE’s criteria.

Thank you very much for your suggestion. The abstract text has been completely rewritten, and the new version has been placed in the manuscript. Additionally, the V4 version has been shortened compared to the V3.

**Introduction**

**Positive:** The introduction clearly outlines the historical development of 3x3 basketball and identifies gaps in the existing literature, highlighting the study's originality and scientific contribution. The research questions are defined clearly and concisely, making it easier for the reader to understand the purpose and significance of the study.

**Negative:**

(0.5) Some parts of the introduction could be long and repetitive. For instance, the sentence **"The characteristics of this sport are defined by the unique dynamics and intensity of the game..."** repeats information already mentioned in the previous sentence. A more concise and focused introduction would help the reader grasp the main idea more quickly.

Following the reviewer's suggestions, the introduction text was rewritten from scratch. Duplicate sentences were removed. The most important aspects of the game were included. The changes also forced an update of the bibliography. In the manuscript, the subchapter was replaced with a new version. Everything was added in V3.

**Materials and Methods**

**Positive:** The methods section provides a detailed explanation of the analytical tools and procedures used. The clear description of the application of the video analysis program and MCDA enhances the study's repeatability and reliability. The level of detail in the methods section demonstrates the scientific rigor of the study.

**Negative:**

(0.6) More information on participant selection and the ethical approval process may be needed. **Specifically, providing further explanation regarding the validity and reliability of the video analysis would strengthen the methodology.** Additionally, it could be beneficial to include more discussion about the advantages and potential limitations of the methods used.

Thank you very much for pointing out the details. The corrections will positively impact the reception of the work.

1. Selection of participants and the ethical consent process: changes introduced in the text.

2. Reliability and credibility of the video analysis: Changes introduced.

3. Advantages and limitations of the methods used: Additional information added.

**Findings**

**Positive:** The results section presents the findings in a detailed and comprehensive manner. The use of tables and graphs to support the data makes it easier for the reader to understand and visualize the results. Notably, the findings highlighting the relationship between shooting strategies and game outcomes clearly demonstrate the main contribution of the study.

**Discussion**

**Positive:** The discussion section compares the findings with the existing literature, highlighting the study's contributions and originality. Statements like "This observation highlights that 2-point shooting training should be a priority for coaches and players..." clearly outline the practical applications of the research and provide recommendations for basketball coaches.

**Negative:**

(0.7) Some expressions in the discussion are repetitive and lengthy. For instance, sentences like "Teams with more effective players with better 2-point shooting accuracy have a greater chance of dominating the pitch" may appear to be a restatement of previous findings. Keeping the discussion more focused and concise would help convey the main conclusions more effectively.

Thank you for your valuable feedback on the discussion section.

In response to your suggestions, we have made the following changes:

1. We have removed repetitions and reduced the number of extended statements to clarify the main conclusions.

2. We have shortened and focused the conclusions, presenting the key points simply and concisely.

3. We have focused on the implications of the results for coaching practice and future research, avoiding unnecessary details.

We hope that the changes we have made meet your expectations and improve the readability of the paper.

**Conclusion**

**Positive:** The conclusion section summarizes the key findings of the research, emphasizing the strategic importance of 2-point shots in 3x3 basketball. Additionally, by offering suggestions on how these findings can be applied in practice, it demonstrates the article's contribution to the field and its potential impact. This can be valuable for coaches and sports analysts.

**Negative:**

(0.8) However, the conclusion could focus more on the study's limitations and the generalizability of the findings. For instance, more information could be provided on how these findings might apply to other basketball variations or different game scenarios. Additionally, suggestions on how the study could be expanded and which aspects should be explored in future research would be beneficial.

Thank you for your valuable feedback on the discussion section.

In response to your suggestions, we have made the following changes:

1. We have added a paragraph highlighting the study's limitations, such as the analysis of only two teams and the specific setting of 3x3 basketball, which may limit the generalizability of the results to other teams and basketball variants.

2. We have suggested potential directions for future research, including extending the analysis to other teams and skill levels, comparisons with 5x5 basketball, and the possibility of using advanced technologies (e.g., LPS tracking systems) to obtain additional data.

**Ethics and Data Sharing**

**Positive:** The article states that ethical standards were followed and data sharing was conducted transparently. This is crucial for the replicability of the study and for it to be re-evaluated by other researchers. Additionally, since the study did not involve human participants or animals, there are no ethical concerns in this regard.

(0.9). As we previously announced, the raw data has been placed in a public repository at [10.5281/zenodo.14021214](https://doi.org/10.5281/zenodo.14021214).
